# CO_2_ Hydrogenation to Methanol over In_2_O_3_ Decorated by Metals of the Iron Triad

**DOI:** 10.3390/molecules29225325

**Published:** 2024-11-12

**Authors:** Tomáš Stryšovský, Martina Kajabová, Arkadii Bikbashev, Zuzana Kovářová, Radka Pocklanová, Robert Prucek, Aleš Panáček, Josef Kašlík, Martin Petr, Libor Kvítek

**Affiliations:** 1 Department of Physical Chemistry, Faculty of Science, Palacký University Olomouc, 17. listopadu 12, 77146 Olomouc, Czech Republic; tomas.strysovsky@upol.cz (T.S.);; 2 Regional Center of Advanced Technologies and Materials, Czech Advanced Technology and Research Institute, Palacký University Olomouc, Šlechtitelů 241/27, 77900 Olomouc, Czech Republic

**Keywords:** iron, cobalt, nickel, indium(III) oxide, methanol synthesis, CO_2_ hydrogenation, heterogeneous catalysis

## Abstract

The growing concentration of CO_2_ in the atmosphere is a serious problem, and efforts to counter this issue are thus highly important. One of the possible approaches to solving this problem is the conversion of waste CO_2_ into products with added economic value. Methanol is one of these products with vast potential usage. In this study, indium oxide prepared by a simple precipitation method and modified by nanoparticles of metals from the iron triad were tested as possible catalysts to produce methanol by the method of CO_2_ hydrogenation. The prepared catalysts demonstrated a strong dependence of their catalytic activity on used metal. The best selectivity for the production of CH_3_OH was observed for the Fe/In_2_O_3_ catalyst at the value of 54.7% at 300 °C. However, due to the higher value of CO_2_ conversion, the highest CH_3_OH formation rate was observed at a value of 11.3 mmol/(h*g) at 300 °C for a composite of Ni/In_2_O_3_.

## 1. Introduction

Anthropogenic CO_2_ is a well-known greenhouse gas and the biggest contributor to global warming, contributing around 60%. The biggest source of this gas is fossil fuel combustion, mainly from the energetics and transport industries. Its emissions are currently so high that the natural carbon cycle can no longer process such high concentrations, which leads to the constant increase in the concentration of this gas in the atmosphere. Therefore, a significant effort is being invested into solving this problem [1,2,3,4,5,6].

Nowadays, two possible routes are available. First is the general decrease of CO_2_ by programs like the EU program for emission reduction, known as the Green Deal [7]. However, the real feasibility of such programs is still the subject of intense discussions. The second possible solution is to capture and recycle waste CO_2_. Two main approaches exist to recycling. The first one is the direct usage of captured CO_2_ (cooling medium, feedstock for algae growth, etc.), and the second is conversion to other chemicals. The most common products of such transformations are CO, methane, methanol, and higher hydrocarbons [8,9].

Direct CO_2_ conversion into methanol has two positive effects: producing a useful compound and reducing its emissions. Methanol can replace gasoline in vehicles and can also be used as solvent and feedstock for the production of more complex compounds (dimethyl ether, formaldehyde, hydrocarbons, acetic acid, etc.). The commercially used catalyst for methanol production by hydrogenation of CO is a composite of copper, zinc(II) oxide, and aluminium(III) oxide. However, commercial catalysts suffer from numerous drawbacks, mainly limited activity and selectivity, which are accompanied by the sintering of an active phase. The development of less problematic catalysts in connection with the utilization of CO_2_ as a raw material for methanol synthesis is thus a popular research topic. The most popular (and promising), are Cu/ZnO-based catalysts, often in combination with ZrO_2_ or other metals and their oxides. However, great attention is also paid to noble metals and In_2_O_3_-based catalysts [8,9,10,11,12,13,14].

Apart from the importance of the carbon-neutral hydrogen source, two main problems are often encountered during CO_2_ hydrogenation. The first problem is the low reactivity of CO_2_ molecules caused by high dissociation energy (1072 kJ/mol). To overcome this, a high reaction temperature (>200 °C) is necessary. The second problem is the exothermic nature of direct methanol synthesis from CO_2_ (Equation (1)). This second problem is amplified by competitive RWGS (reverse water gas shift) reaction, which is endothermic (Equation (2)) [5,10,12,15].

CO_2_ + 3H_2_ ↔ CH_3_OH + H_2_O    _∆_H_25_ = −49.5 kJ/mol (1)

CO_2_ + H_2_ ↔ CO + H_2_O              _∆_H_25_ = +41.2 kJ/mol (2)

Competition between these two reactions leads to a situation, where the methanol synthesis is kinetically limited at lower temperatures and thermodynamically at higher temperatures. Both problems can be solved by using indium oxide-based catalysts. The reason is the activation energy of methanol synthesis using this catalyst (103 kJ/mol), which is lower than that of RWGS (117 kJ/mol) at typical conditions for methanol synthesis (pressure about 5 MPa and reaction temperature between 200 and 300 °C) [12,16,17,18,19].

This study focused on the effect of metals of the iron triad on the catalytic activity of indium oxide support. All three metals are commonly used in heterogeneous catalysis, e.g., in CO_2_ hydrogenation to produce methane or higher hydrocarbons. However, only Ni is commonly used as a part of methanol-producing catalysts, e.g., Ni/In_2_O_3_, Ni/β-Ga_2_O_3_, or M-Ni-Ga/SiO_2_ (M = Au, Cu, Co) [20,21,22,23,24,25,26,27]. Thus, our goal was to evaluate the activity of these metals (or their oxides) in a less traditional role of the methanol-producing catalysts. In_2_O_3_ was prepared using the precipitation method followed by calcination. Fe, Co, and Ni nanoparticles were subsequently immobilised by ultrasound-assisted reduction of an appropriate metal salt. Catalytic activity tests were performed in a fixed-bed continuous flow microreactor at a total reaction mixture pressure of 3 MPa. All tested catalysts were active with CO, methanol, and methane as main products. All catalysts also catalysed the production of dimethyl ether, but only in trace amounts.

## 2. Results

### 2.1. Catalysts Properties

**Figure 1:**
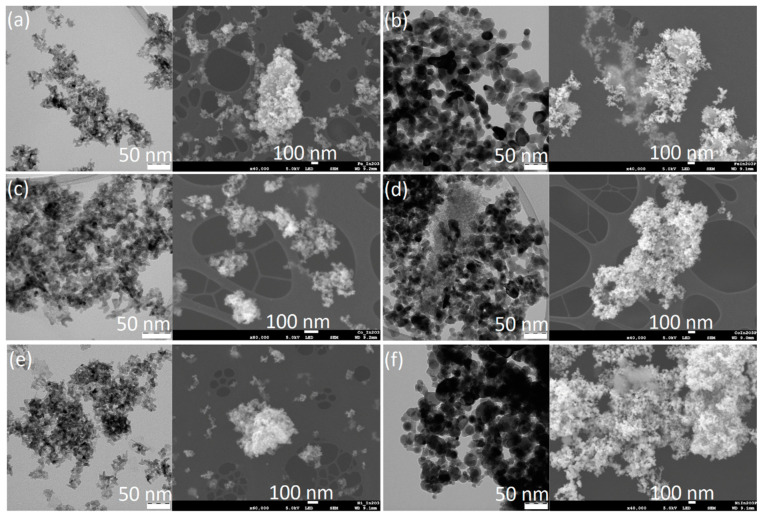
TEM and SEM images of fresh and spent catalysts. Fe/In_2_O_3_: (**a**) fresh, (**b**) spent. Co/In_2_O_3_: (**c**) fresh, (**d**) spent. Ni/In_2_O_3_: (**e**) fresh, (**f**) spent.

All three prepared catalysts were crystalline powders. XRD patterns (Figure S1) of Ni and Co-decorated catalysts were identical, without the detectable presence of Ni and Co. Particles of these metals were probably too small to be detectable by XRD. Fe in Fe-decorated catalysts was detectable and present in the compound identified as Fe_0.2_In_1.8_O_3_ (PDF-4+ database, card 04-017-4223). Indium oxide was present in the cubical morphology structure (PDF-4+ database, card 04-008-2022). All samples’ morphology and particle sizes (average 7–8 nm) were identical. However, catalysts differed in colour. Colours were brown (Fe/In_2_O_3_), brown-green (Co/In_2_O_3_), and green-yellow (Ni/In_2_O_3_) (Figure S2). Both TEM and SEM images are presented in Figure 1a,c,e. Exact metal loadings determined by AAS were 4.60 *w*/*w*% for Fe, 4.78 *w*/*w*% for Co and 4.82 *w*/*w*% for Ni. While the H_2_-TPR spectra (Figure S3) of Fe/In_2_O_3_ and Co/In_2_O_3_ were similar to each other, the spectrum of Ni/In_2_O_3_ was slightly different. Two main peaks were observable in all spectra; their exact position depends on the metal used. The first peak [~192 °C (Fe), ~212 °C (Co), and ~202 °C (Ni)] shows the effect of the used metal on the reducibility of the In_2_O_3_ surface. The second peak [~440 °C (Fe) and ~398 °C (Co)] is more complex in the case of Ni because it is composed of two overlapping peaks (~335 °C and ~471 °C). This region is usually considered an area of surface metal reduction (Fe, Co, Ni) accompanied by In-X alloy formation (alloy presence was confirmed by XPS analysis of the spent catalysts), so two peaks in the case of Ni could mean the formation of more complex alloys caused by strong interactions on the Ni-In_2_O_3_ interface. The overall intensity of this peak suggests that these interactions are much stronger than on the Fe- and Co-In_2_O_3_ interfaces. The steep increase in signal starting at ~450 °C with a maximum range of graphs is the reduction of In_2_O_3_ bulk. CO_2_-TPD (Figure S4) spectra of catalysts were also similar. Low-temperature peaks [~87 °C and ~177 °C (Fe), ~86 °C (Co), and ~101 °C (Ni)] represent the desorption of weakly bonded physisorbed CO_2_. The medium temperature peak exclusive for Fe is the desorption of moderately strongly bonded CO_2_. The high-temperature peak [~466 °C (Fe), ~485 °C (Co) and ~461 °C (Ni)] is desorption from the oxygen vacancies [28]. The relatively low intensity of low-temperature peaks in the spectrum of Fe/In_2_O_3_ could be a possible explanation for observed low conversions.

**Figure 2:**
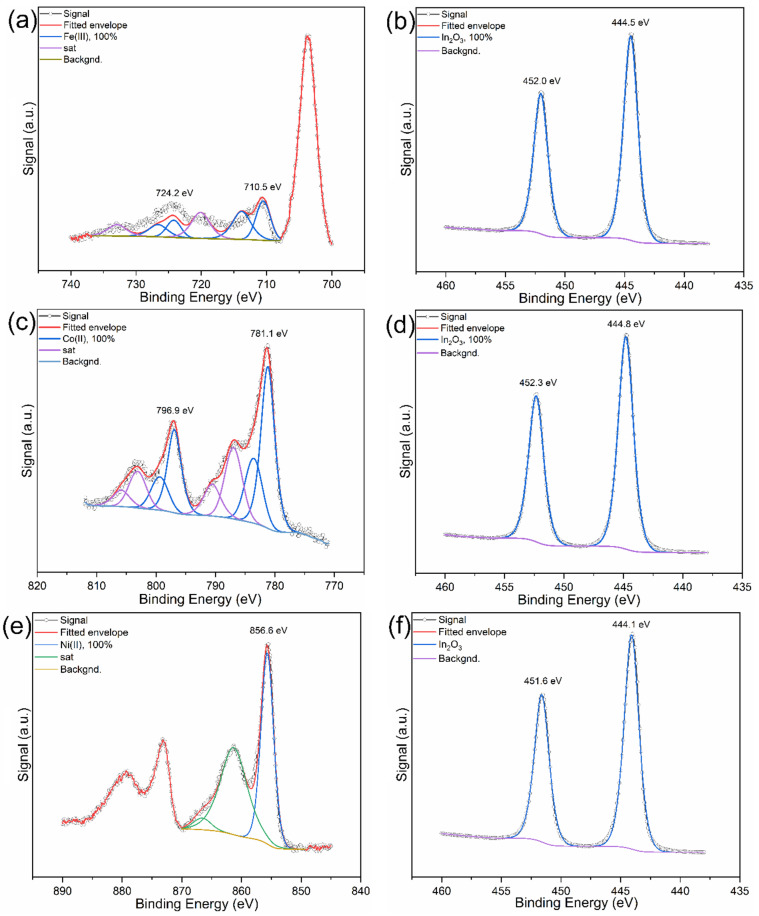
XPS spectra of (**a**) Fe 2p and (**b**) In 3D regions of fresh Fe/In_2_O_3_; (**c**) Co 2p and (**d**) In 3D regions of fresh Co/In_2_O_3_; (**e**) Ni 2p and (**f**) In 3D regions of fresh Ni/In_2_O_3_.

The XPS spectra are shown in Figure 2. Fe, Co, and Ni are all found in their oxidised states, with Fe(III), Co(II), and Ni(II) species present, indicated by binding energies of their main peaks at 710.5 eV for Fe 2p, 781.1 eV for Co 2p, and 856.6 eV for Ni 2p. The indium(III) oxide support is also clearly visible in each sample with a characteristic In 3D binding energy around 444.5 eV, confirming its presence as the dominant indium phase. Each metal appears to be fully oxidised and In_2_O_3_ shows no sign of degradation or phase transformation, maintaining a 100% presence in all samples.

### 2.2. Catalytic Activity

The main products of catalysed hydrogenation of CO_2_ in the presence of prepared catalysts were carbon monoxide, methanol, and methane. The secondary product was dimethyl ether, but the produced amount was neglectable. The tested catalysts demonstrated a strong dependence of their catalytic activity on used dopants.

**Figure 3:**
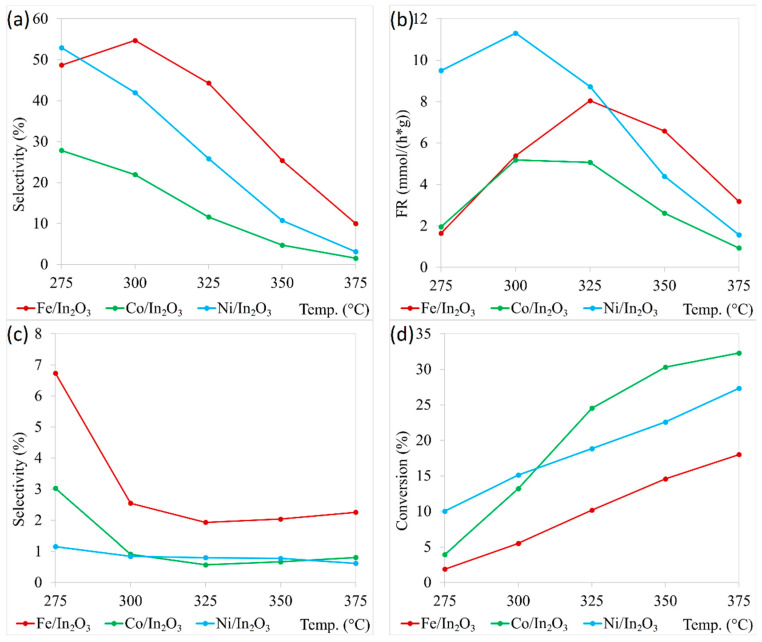
Catalytic activity of prepared catalysts: (**a**) CH_3_OH selectivity and (**b**) FR, (**c**) CH_4_ selectivity, and (**d**) CO_2_ conversion.

The general shape of the curves for all the observed characteristics was the same for all catalysts. The difference was in the obtained values and temperatures of optimal performance. All catalysts achieved the highest conversions at the highest tested temperature of 375 °C. Values were 18% for Fe/In_2_O_3_, 27.3% for Ni/In_2_O_3,_ and 32.3% for Co/In_2_O_3_. High conversion achieved with Co/In_2_O_3_ was caused mainly by high production of CO. Activity of this catalyst toward the production of methanol was subpar in comparison with the remaining two catalysts (the highest selectivity 27.9% at 275 °C). The best methanol selectivity demonstrated catalyst Fe/In_2_O_3_, with a maximum of 54.7% at 300 °C. However, this catalyst showed the lowest conversion, which caused that catalyst Ni/In_2_O_3_ to have better FR of methanol—8 mmol/(h*g) at 325 °C for Fe/In_2_O_3_ versus 11.3 mmol/(h*g) for Ni/In_2_O_3_ at 300 °C. The activity of all the tested catalysts towards the production of methane was very low (the highest selectivity ~6.7% at 275 °C), and the produced amounts were neglectable in comparison with other products due to a steep decrease in selectivity at higher temperatures down to 1–2% for all tested catalysts. All data are graphically presented in Figure 3 and Figure S5.

### 2.3. Spent Catalysts Characterisations

**Figure 4:**
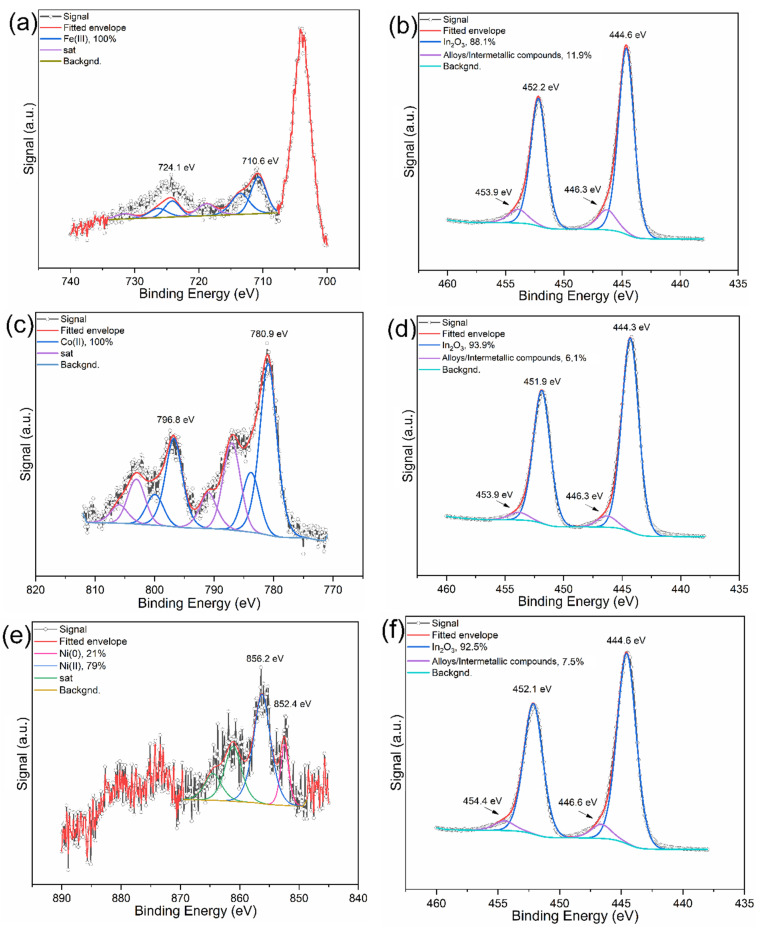
XPS spectra of (**a**) Fe 2p and (**b**) In 3D regions of spent Fe/In_2_O_3_; (**c**) Co 2p and (**d**) In 3D regions of spent Co/In_2_O_3_; (**e**) Ni 2p and (**f**) In 3D regions of spent Ni/In_2_O_3_.

TEM and SEM images (Figure 1b,d,f) showed that exposure of the catalysts to the reaction conditions led to an increase in particle size [19.9 nm (Fe), 14.3 nm (Co) and 24.1 nm (Ni)]. However, the general morphology remained similar to the fresh catalysts. Also, XRD analysis did not reveal any fundamental changes in the structure and composition of the tested catalysts (Figure S6). Patterns of spent Fe- and Co-decorated catalysts remained almost identical to those of their fresh versions. The only notable difference was the presence of an amorphous phase. In the case of Ni-decorated catalysts, the presence of NiO was detected (PDF-4+ database, card 04-023-3539), which was not observed for fresh catalysts. The absence of In^0^ suggests a low level of In^III^ reduction. This was confirmed also by XPS analysis (Figure 4). The XPS data after catalysis indicated notable changes in the oxidation states of Ni and a slight modification in the indium oxide support. Cobalt remained in the Co(II) state, with a minor shift in binding energy to 780.9 eV, while iron retained its Fe(III) state at 710.6 eV, showing no significant reduction. However, nickel undergoes partial reduction, with a new Ni(0) signal at 852.4 eV, representing 21% of the total nickel signal, while the remaining 79% persists as Ni(II) at 856.2 eV. This partial reduction of nickel suggests that catalysis has a stronger reductive impact on Ni compared to Co and Fe, likely due to its relatively lower reduction potential. The In_2_O_3_ support also shows minor transformation, seen as a secondary indium peak near 446.3–446.6 eV, indicating a new indium compound (alloy or intermetallic compound). This additional peak, constituting 6.1–11.9% of the indium signal across the samples, suggests a slight alteration in the chemical environment.

## 3. Discussion

The results obtained from the catalytic activity of the prepared composite catalysts showed the strong influence of selected dopants on their catalytic activity in CO_2_ hydrogenation. The production of all three main products and the degree of CO_2_ conversion was influenced by this factor.

All the tested catalysts achieved the highest conversion at the highest tested temperature. The increase in conversion for Ni- and Fe-decorated catalysts was relatively linear, but the ability of Ni to catalyse the splitting of CO_2_ was significantly higher (27.3% vs. 18% conversion). The profile of this curve for Co-decorated catalysts was different, with a rapid increase in conversion at lower temperatures and a much slower increase at higher temperatures. This catalyst also achieved the highest conversion of all three tested catalysts. However, high conversion in the presence of this catalyst is caused by very high activity toward RWGS reaction and, thus, high production of CO.

All tested catalysts were active toward the production of methanol. Unsurprisingly, the best overall activity demonstrated catalyst Ni/In_2_O_3_ with the highest achieved FR with 11.3 mmol/(h*g) value. However, this catalyst was surpassed by Fe/In_2_O_3_ in methanol selectivity. Except for the lowest tested temperature, Fe/In_2_O_3_ demonstrated much higher selectivity than Ni/In_2_O_3_. However, lower conversions caused lower FR of methanol at the three most interesting temperatures (275 °C, 300 °C, and 325 °C). The activity of Co/In_2_O_3_ toward methanol (both selectivity and FR) was subpar to the other tested catalysts, despite high conversions. The activity of the tested Ni and Co-modified catalysts is comparable with already published results (Table 1). The presented results confirm the high catalytic activity of Ni-modified In_2_O_3_ toward methanol formation, which is generally comparable with the activity of the noble metal-modified indium oxide catalyst. This is a positive finding for the development of a cheap catalyst with high performance for such an interesting reaction as CO_2_ hydrogenation.

Apart from CO and methanol, methane and dimethyl ether were other products. Production of methane was low for all the tested catalysts. Selectivity for this gas was the highest at the lowest tested temperature, but suddenly, it dropped and remained relatively stable at all remaining temperatures. This is interesting because Co and Fe are commonly used as methane production catalysts, and selectivity below 3% during most tested temperatures is unimpressive [36,37]. Dimethyl ether was produced only in trace amounts and only in temperatures ranging from 300 °C to 350 °C.

It is worth noting that (apart from particle size) the composition and morphology of all catalysts remained mostly unchanged during the catalytic tests. However, this is not valid for surface composition. The results of the XPS study of the spent catalysts suggest that the composition of the surface was changed by the formation of X-In (X = Fe, Ni, or Co) intermetallic compounds or alloys. This is in sharp contrast with our previous study focused on Cu/In_2_O_3_ catalysts. These catalysts were significantly reduced, and the presence of alloys was detectable also by XRD. However, the different behaviour of Cu-modified catalysts could be caused by the different reaction conditions used in this study [19]. On the other hand, XPS analysis did not confirm any reduction of In_2_O_3_ to In^0^ although H_2_-TPR analysis suggests that surface layers of In_2_O_3_ should be at least partially reduced to In^0^. Thus, surface layers are probably reoxidized during the reaction. Degrees of reduction of Ni^II^, Co^II^, and Fe^III^ were also very low, where only Ni^II^ was at least partially reduced to metal. The Ni^0^ particles provide the optimal surface for H_2_ dissociation, which is an important step for the catalytic hydrogenation of CO_2_ [30]. The same can be assumed for the action of Co particles [24]. However, as Fe^0^ was not observed in the XPS spectra of the spent Fe/In_2_O_3_ catalyst, a different mechanism is probably involved in the process of the methanol formation on this catalyst. However, a lack of published studies about this type of catalyst in the literature shows the need to focus research on this catalyst composition to attain the best results in the field of methanol production via hydrogenation of CO_2_.

## 4. Materials and Methods

### 4.1. In_2_O_3_ Synthesis

Indium(III) oxide was synthesised using the precipitation method followed by calcination. In total, 3 g of In(NO_3_)_3_·xH_2_O (Sigma-Aldrich, St. Louis, MO, USA, 99.99%) was dissolved in 39 mL of deionised water. A solution of 3.9 g of Na_2_CO_3_ (Lach-Ner, Neratovice, Czech Republic, p.a.) in 46.8 mL of water was slowly added under constant stirring. Subsequently, the total volume of solution was increased to 155 mL by the addition of the pure water, and the pH was adjusted to ~9.2 by dropwise addition of diluted HNO_3_. After the formation of a precipitate, the solution was aged for one hour at laboratory temperature. The precipitate was then separated by centrifugation, washed, and dried overnight at 60 °C. The dried precipitate was calcined at 300 °C for 3 h (heating rate ~3.5 °C/min).

The prepared In_2_O_3_ was modified with either Fe, Co, or Ni by ultrasound-assisted reduction of an appropriate salt by NaBH_4_ (Sigma-Aldrich, St. Louis, MO, USA, ≥98%) solution, as was successfully used in our other studies [38,39]. The preparation procedure for all three metals was identical: 1 g of indium(III) oxide was added to solution of metal salt [262.12 mg of FeSO_4_·7H_2_O (Lach-Ner, Neratovice, Czech Republic, p.a.), 259.9 mg of Co(NO_3_)_2_·6H_2_O (Lach-Ner, Neratovice, Czech Republic, p.a.), or 213.14 mg of NiCl_2_·6H_2_O (Chemapol, Prague, Czech Republic, pure)] in 100 mL of water. Additionally, a solution of NaBH_4_ (71.3 mg for Fe, 67.57 mg for Co, and 68 mg for Ni) in 100 mL was prepared and aged for 20 min. The prepared solution was rapidly added to the In_2_O_3_ aqueous dispersion with added metal ions. The mixture was sonicated and stirred throughout. The total length of sonication was 10 min at a power of 30 W. The prepared composites were collected by centrifugation, washed, and dried overnight at 60 °C. The theoretical metal loading was 5 *w*/*w*% in all three cases.

### 4.2. Characterization Methods

Scanning electron microscopy (SEM) Jeol-7900F (JEOL, Tokyo, Japan) and transmission electron microscopy (TEM) JEM-2100 (JEOL, Tokyo, Japan) were used to determine the morphology and structure of the samples. Particle sizes were determined from TEM images using ImageJ software v1.52a. Surface composition was determined by an X-ray photoelectron spectroscopy (XPS) Nexsa G2 XPS system (Thermo Fisher Scientific, Waltham, MA, USA) with monochromatic Al-Kα source and photon energy of 1486.7 eV. All the spectra were measured in the vacuum of 1.2*10^−7^ Pa and at a room temperature of 20 °C. The high-resolution spectra were measured with a pass energy of 30.00 eV and an electronvolt step of 0.1 eV. Charge compensation was used for all measurements. The spectra were evaluated with the Advantage 6.5.1 (Thermo Fisher Scientific, Waltham, MA, USA) software. Phase compositions were identified by X-ray diffractometer X’Pert PRO MPD (Malvern Panalytical, Malvern, United Kingdom) with a Co anode and scanning angle 2Θ from 5 to 105°. PDF-4+ database was used for the pattern analyses. A 3Flex Adsorption Analyzer (Micromeritics, Norcross, GA, USA) was used for measurement of H_2_-TPR and CO_2_-TPD spectra in the range from 40 °C to 600 °C (temperature ramp 10 °C/min). For CO_2_-TPD, samples were activated in the flow of He (22.5 mLN/min) at 300 °C for 1 h. After activation, the samples were cooled to the laboratory temperature and left under the flow of CO_2_ (50 mLN/min) for 30 min. Measurement was performed under the flow of 50 mLN/min He. For H_2_-TPR, the samples were not pretreated and the reaction mixture of 10 vol% H_2_ in Ar (50 mLN/min) was used. The weight of the sample was around 50 mg for both analysis types. The exact metal loadings were determined by Analytic Jena contrAA 300 AAS (Analytic Jena, Jena, Germany).

### 4.3. Activity Test

Catalytic testing was performed in a microreactor Microactivity Effi (PID Eng&Tech, Madrid, Spain). In total, 100 mg of catalyst diluted by 150 mg of silica (Silica for GC chromatography, Penta, Prague, Czech Republic) was loaded in a steel capillary with a diameter of 5.1 mm. The catalysts were activated at 300 °C for 1 h at a flow of 22.5 mLN/min of He and a pressure of 5 bar. Catalysts were tested at temperatures from 275 °C to 375 °C with a temperature ramp of 25 °C. The catalysis was performed at each temperature for 3 h. The He/H_2_/CO_2_ molar ratio was 5:76:19 with a total gas hourly space velocity of 21,000 mLN/(h*gcat), and a pressure of 30 bar. Spent catalysts were stored in a glove box under N_2_ inert atmosphere to prevent their reoxidation in air.

The products were determined and quantified by gas chromatograph Agilent 7890B GC (Agilent Technologies, Santa Clara, CA, USA) coupled with mass spectrometer Agilent 5977B (Agilent Technologies, Santa Clara, CA, USA) as a detector. Columns Shin Carbon 100/120 (1 m; 1 mm) (Restek, Centre County, PA, USA) and PoraBOND QPT (25 m; 0.32 mm; 5 µm) (Agilent Technologies, Santa Clara, CA, USA) were used for the analysis of gaseous products. The products were analysed at hourly intervals. Conversion of CO_2_ (*x_CO2_*), product selectivity (*s_x_*) and formation rate (*FR*) were calculated according to Equations (3)–(5):

(3) $$x_{{CO}_{2}}\left(\%\right)=\left(1-\frac{Pala}{\left[{CO}_{2}\right]+\left[CO\right]+\left[{CH}_{4}\right]+\left[{CH}_{3}OH\right]}\right)*100$$

(4) $$s_{x}\left(\%\right)=\left(\frac{\left[X\right]}{\left[CO\right]+\left[{CH}_{4}\right]+\left[{CH}_{3}OH\right]}\right)*100$$

(5) $${FR}_{x}=\left(\frac{F_{{CO}_{2}}*x_{{CO}_{2}}*S_{x}}{m_{cat}}\right)*1000$$

where *FR* (formation rate) expresses the amount of product in mmol per hour and per gram of catalyst [mmol/(h*g_cat_)]. $F_{{CO}_{2}}$ expresses the flow of CO_2_ per hour (in moles) and *m_cat_* represents the weight of the catalyst. [*X*] is a molar concentration.

## 5. Conclusions

Iron triad metals were used for modifying indium oxide-based catalysts and were studied for CO_2_ hydrogenation. Catalysts were prepared by ultrasound-assisted immobilisation of Fe, Co, and Ni nanoparticles on pre-prepared indium(III) oxide. In_2_O_3_ was prepared using the precipitation method followed by calcination.

The catalytic studies demonstrated good catalytic performance of all iron triad metals as dopants for indium oxide-based methanol reformation catalysts. Ni confirmed itself as the most suitable dopant from the point of view of the formation rate of methanol, while Fe showed the best selectivity for methanol production, which was higher than in the case of Ni. Therefore, Fe-decorated In_2_O_3_ could be a potential candidate for further optimisation in its composition to improve the whole catalytic process. An effort to increase CO_2_ conversion will be vital, which is the biggest drawback of tested catalysts. The worst performance toward methanol showed a Co-decorated catalyst. However, a combination of high CO selectivity and high CO_2_ conversion could be beneficial for syngas-production catalysts.

## Figures and Tables

**Table 1:** Comparisons with other catalysts.

Catalyst	Pressure (bar)	GHSV (mL/(g_cat_*h))	Temperature (°C)	CO_2_ Conversion (%)	CH_3_OH Selectivity (%)	STY (g_met_/(g_cat_*h))	Reference
Ni/In_2_O_3_	50	21,000	300	18.47	55	0.55	[28]
Ni/In_2_O_3_	50	9000	300	~11.5	~46	~0.16	[29]
Co/In_2_O_3_	50	9000	300	~8	~57	~0.13	[29]
NiO(6)-In_2_O_3_	30	60,000	250	~3	53	0.26	[30]
NiO(6)-In_2_O_3_	30	60,000	300	---	27	0.41	[30]
In_1_-Co_4_	40	24,000	300	8.9	46.5	0.31	[31]
Au/In_2_O_3_	50	21,000	300	11.7	67.8	0.47	[32]
Pt/In_2_O_3_	50	21,000	300	17.6	54	0.54	[33]
Cu/In_2_O_3_	30	7500	280	11.4	80.5	0.197	[34]
Au/In_2_O_3_	50	24,000	280	~1.9	56	0.07	[35]
Co/In_2_O_3_	50	24,000	280	4	72	0.2	[35]
Ni/In_2_O_3_	50	24,000	280	~6.1	75	0.31	[35]
Fe/In_2_O_3_	30	21,000	325	10.2	44.3	0.26	This work
Co/In_2_O_3_	30	21,000	300	13.2	21.9	0.17	This work
Ni/In_2_O_3_	30	21,000	300	15.1	42	0.36	This work

## Data Availability

Dataset available on request from the authors.

## Supplementary Materials

The following supporting information can be downloaded at: https://www.mdpi.com/article/10.3390/molecules29225325/s1, Figure S1: XRD patterns of fresh (a) Fe/In_2_O_3_, (b) Co/In_2_O_3_ and (c) Ni/In_2_O_3_; Figure S2: Images of fresh (a) Fe/In_2_O_3_, (b) Co/In_2_O_3_ and (c) Ni/In_2_O_3_; Figure S3: H_2_-TPR spectra of (a) Fe/In_2_O_3_, (b) Co/In_2_O_3_ and (c) Ni/In_2_O_3_; Figure S4: CO_2_-TPD spectra of (a) Fe/In_2_O_3_, (b) Co/In_2_O_3_ and (c) Ni/In_2_O_3_; Figure S5: Catalytic activity of prepared catalysts: (a) CO selectivity and (b) FR, (c) CH_4_ FR; Figure S6: XRD patterns of spent (a) Fe/In_2_O_3_, (b) Co/In_2_O_3_ and (c) Ni/In_2_O_3_.
