# Supplementary material for: CO2 Hydrogenation to Methanol over In2O3 Decorated by Metals of the Iron Triad
Source: Molecules. 2024 Nov 12;29(22):5325. doi: 10.3390/molecules29225325 (PMC11597088; doi:10.3390/molecules29225325)
Supplement: Supplementary file 1 [file molecules-29-05325-s001.zip › molecules-3242750-supplementary.pdf]

CO_2 hydrogenation to methanol over In_2O_3 decorated by metals of iron triad

Tomáš Stryšovský [1], Martina Kajabová[1], Arkadii Bikbashev[1], Zuzana Kovářová[1], Radka Pocklanová[1], Robert Prucek [1], Aleš Panáček [1] Josef Kašlík [2], Martin Petr[2] and Libor Kvítek [1,*]

1 Department of Physical Chemistry, Faculty of Science, Palacký University Olomouc, 17. listopadu 12, CZ-77146 Olomouc, Czech Republic
2 Regional Center of Advanced Technologies and Materials, Czech Advanced Technology and Research Institute, Palacký University Olomouc, Šlechtitelů 241/27, CZ-77900 Olomouc, Czech Republic

* Correspondence: libor.kvitek@upol.cz; Tel.: + 420 585 634 420

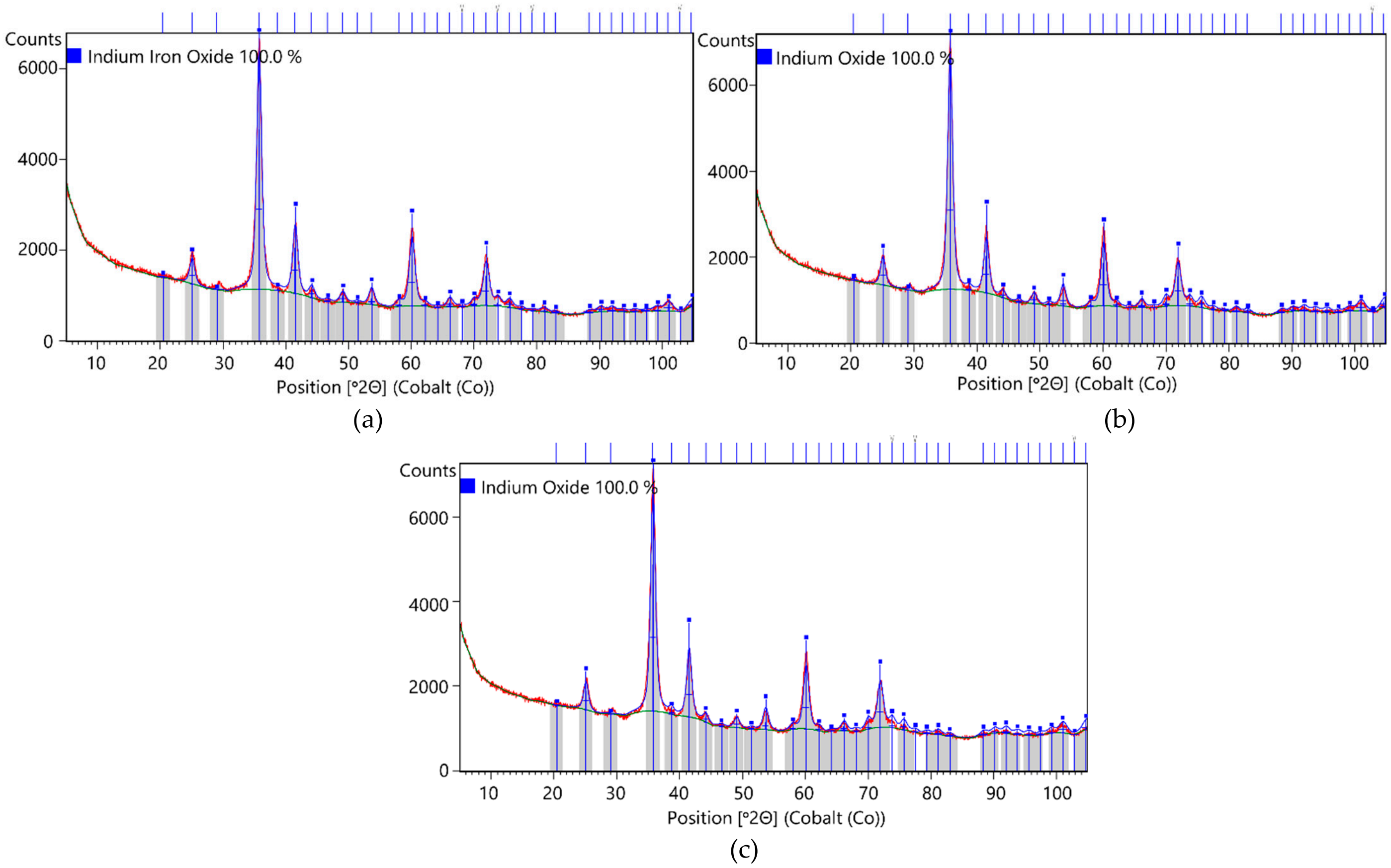

Figure S1: XRD patterns of fresh (a) Fe/In_2O_3, (b) Co/In_2O_3 and (c) Ni/In_2O_3.

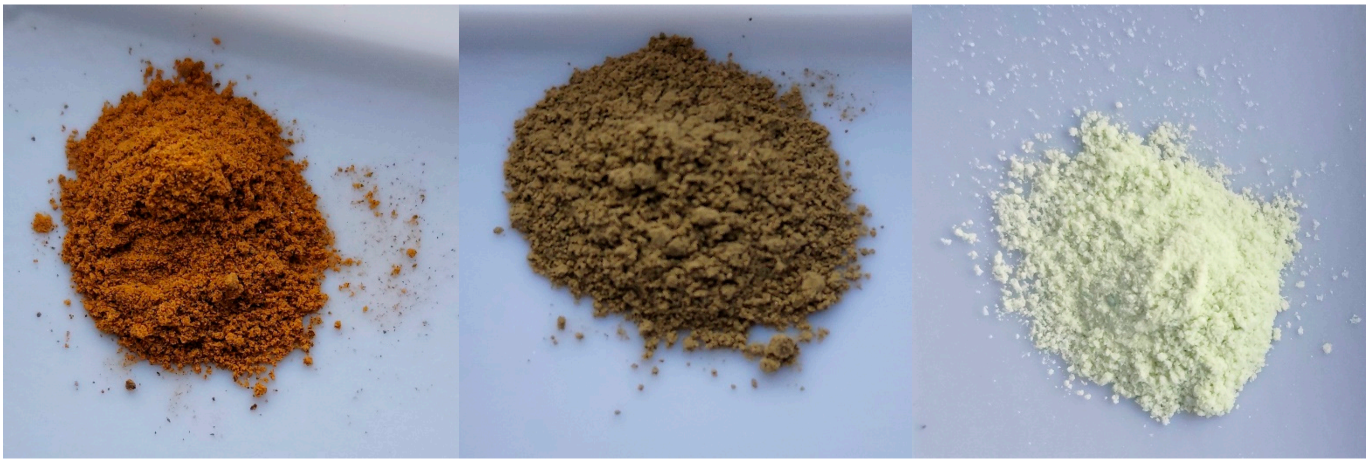

(a) (b) (c)

Figure S2: Images of fresh (a) Fe/In_2O_3, (b) Co/In_2O_3 and (c) Ni/In_2O_3.

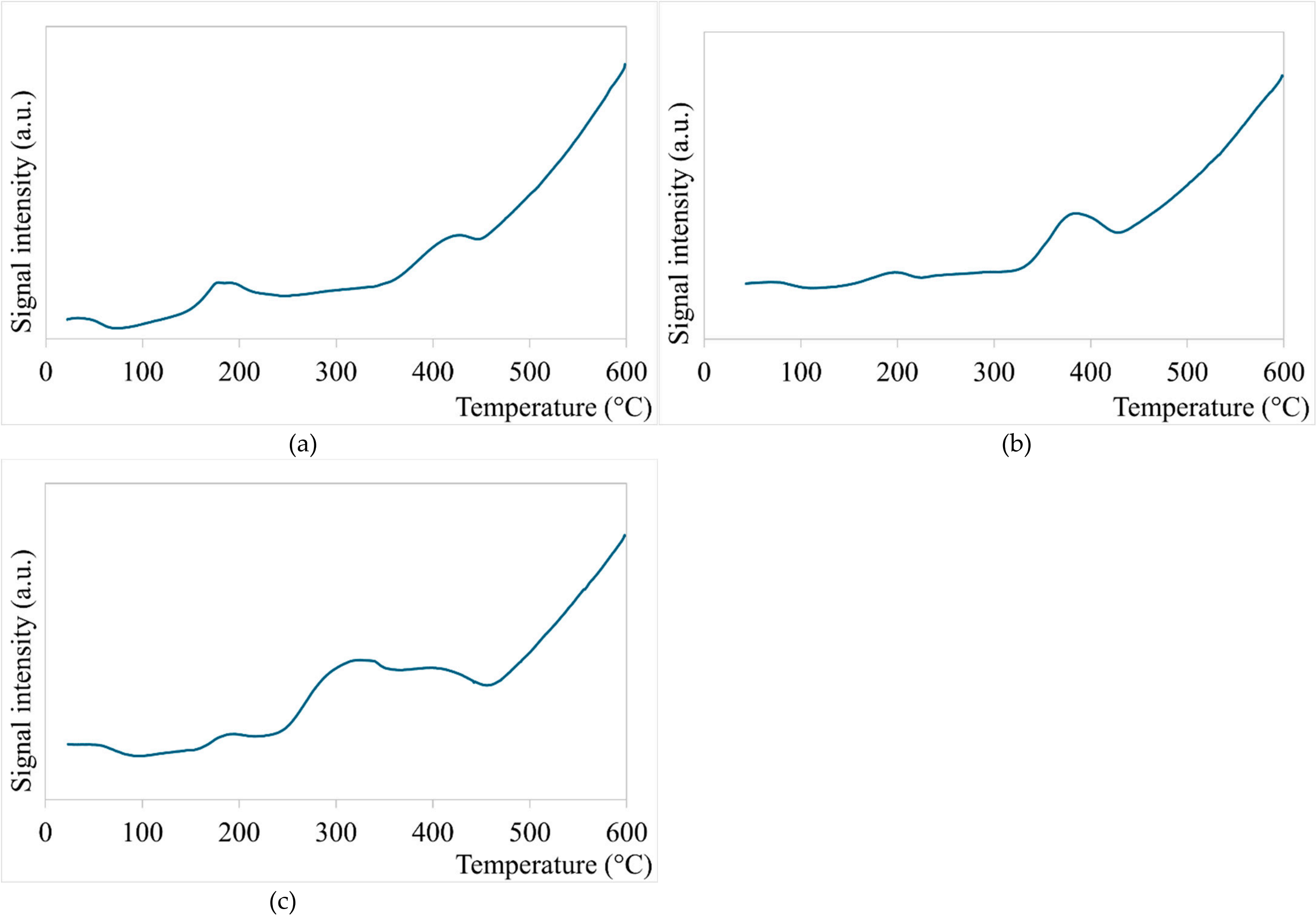

Figure S3: H_2-TPR spectra of (a) Fe/In_2O_3, (b) Co/In_2O_3 and (c) Ni/In_2O_3.

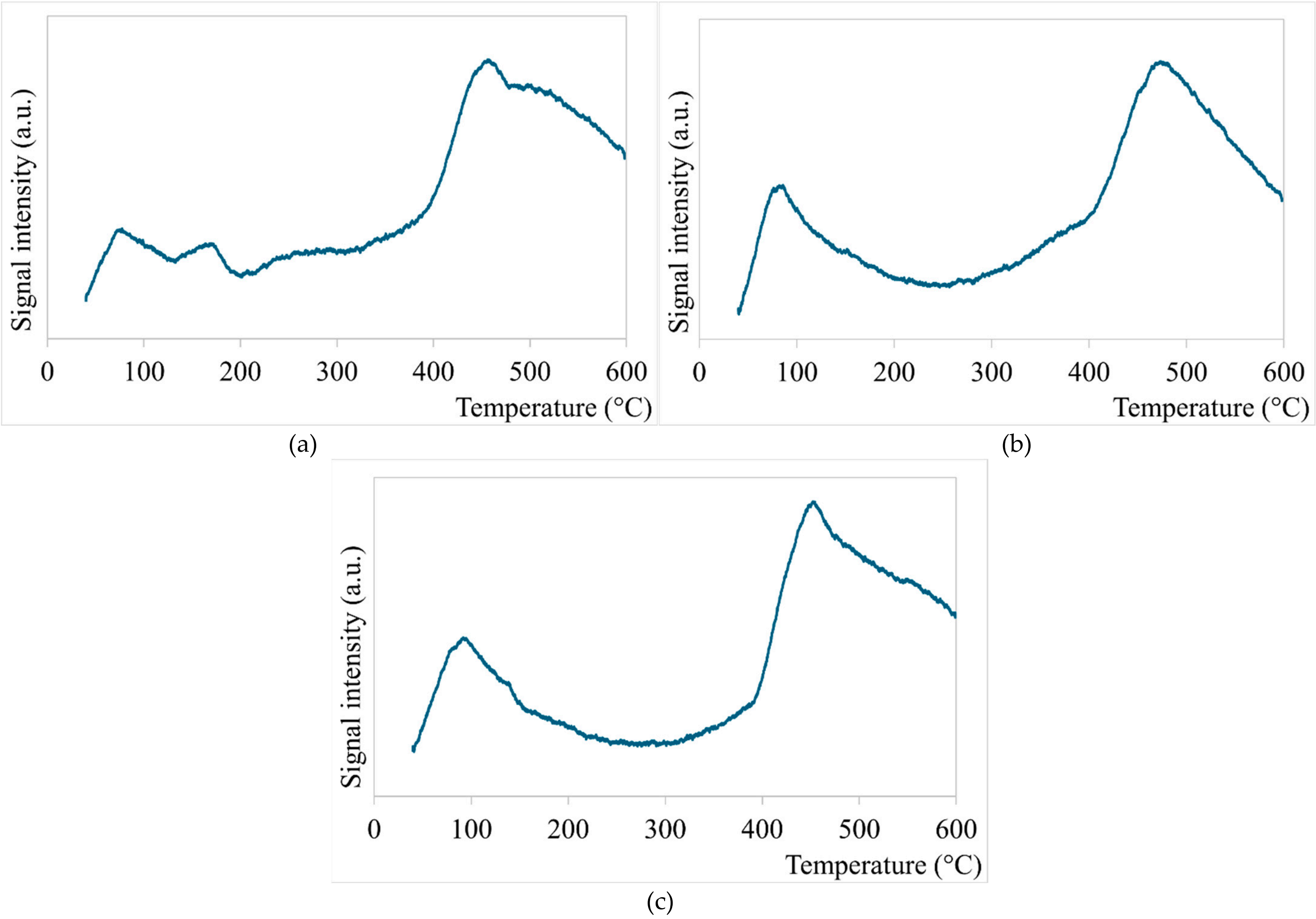

Figure S4: CO_2-TPD spectra of (a) Fe/In_2O_3, (b) Co/In_2O_3 and (c) Ni/In_2O_3.

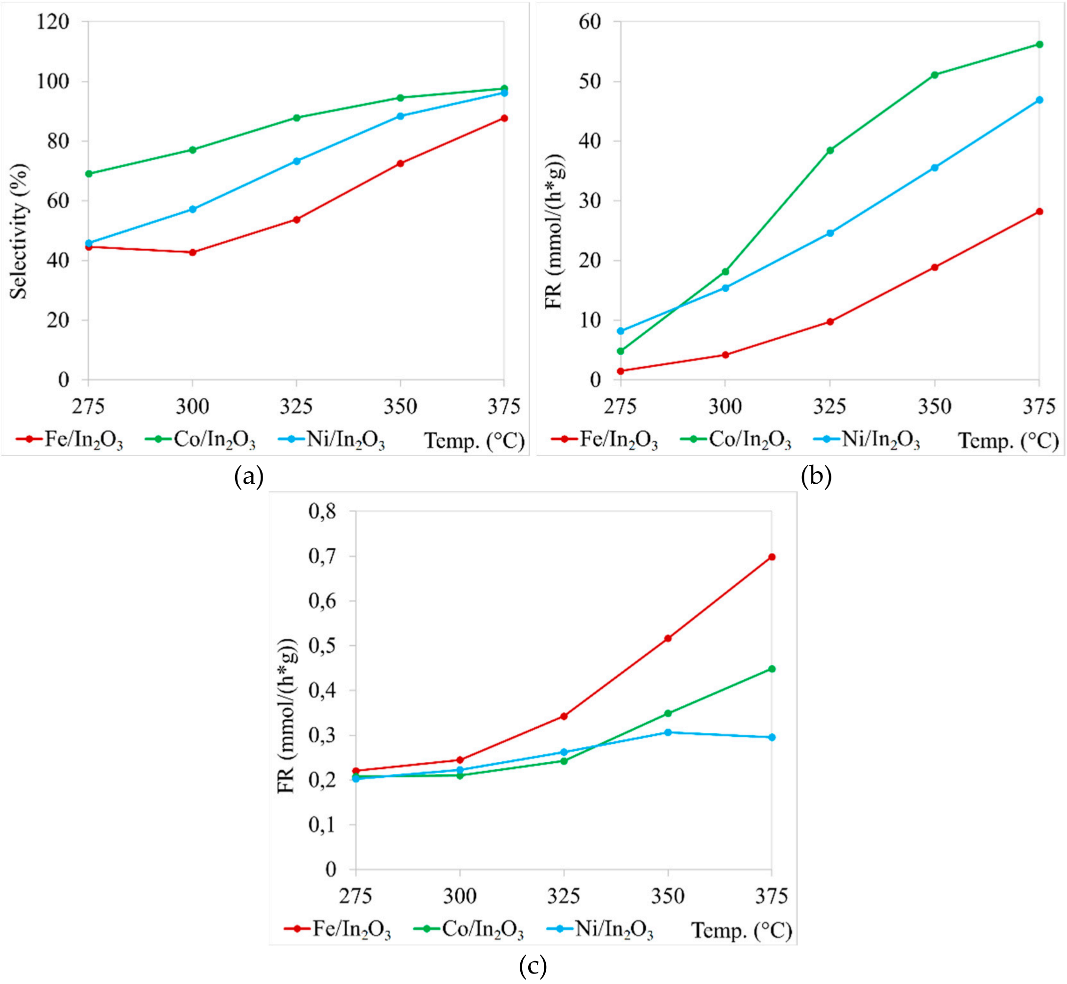

Figure S5: Catalytic activity of prepared catalysts: (a) CO selectivity and (b) FR, (c) CH_4 FR.

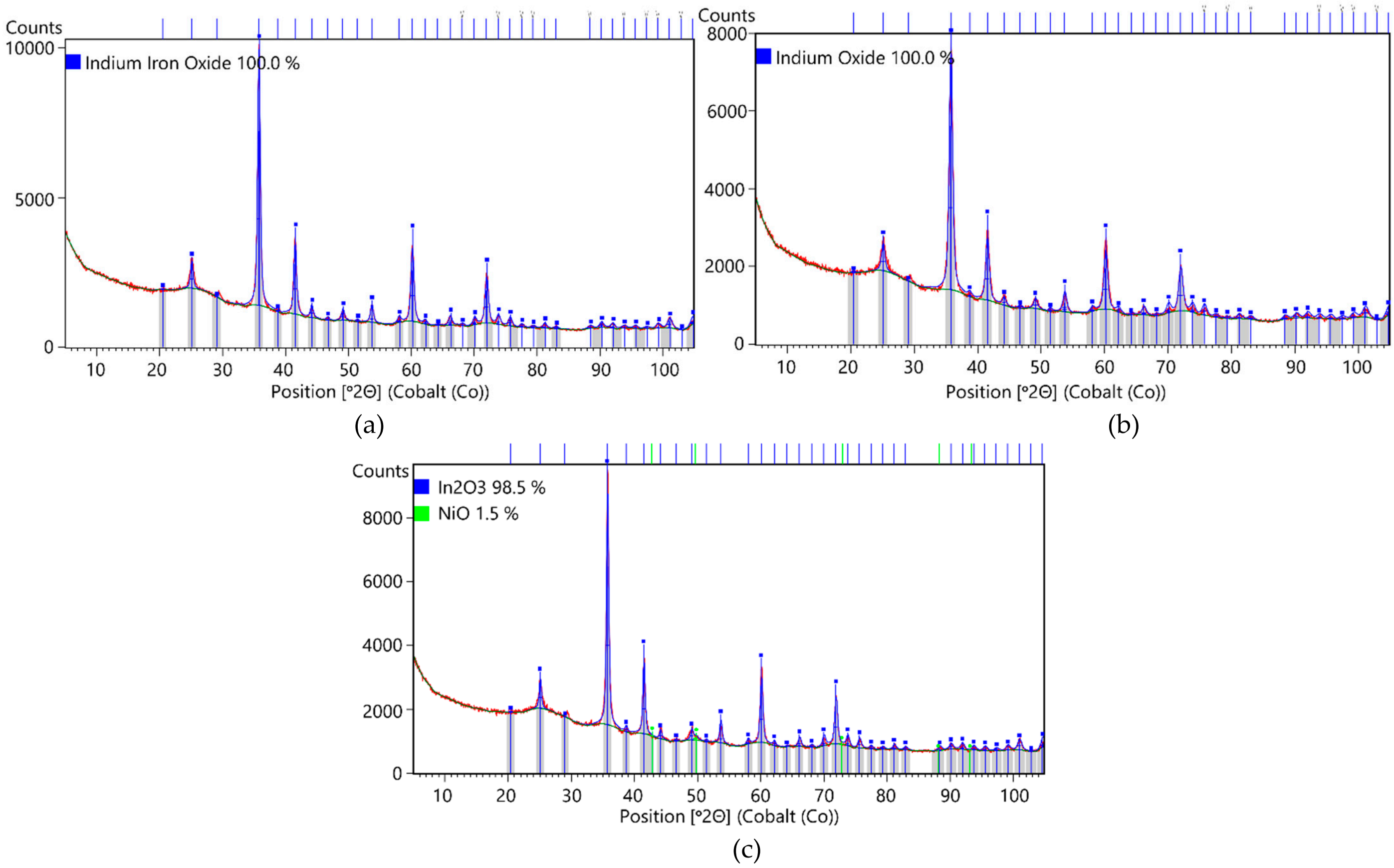

Figure S6: XRD patterns of spent (a) Fe/In_2O_3, (b) Co/In_2O_3 and (c) Ni/In_2O_3.
